# Unveiling the Antifungal Potential of Two Iberian Thyme Essential Oils: Effect on *C. albicans* Germ Tube and Preformed Biofilms

**DOI:** 10.3389/fphar.2019.00446

**Published:** 2019-05-02

**Authors:** Melissa Alves, Maria José Gonçalves, Mónica Zuzarte, Jorge M. Alves-Silva, Carlos Cavaleiro, Maria Teresa Cruz, Lígia Salgueiro

**Affiliations:** ^1^CIEPQPF – Department of Chemical Engineering and Faculty of Pharmacy, University of Coimbra, Coimbra, Portugal; ^2^Faculty of Medicine, CNC.IBILI, University of Coimbra, Coimbra, Portugal; ^3^Faculty of Pharmacy, CNC.IBILI, University of Coimbra, Coimbra, Portugal

**Keywords:** *Thymus camphoratus*, *Thymus carnosus*, essential oil, germ tube, biofilm, cytotoxicity

## Abstract

Fungal infections remain a burden worldwide, thus underpinning the need for effective new therapeutic approaches. In the present study, the antifungal effect of the essential oils of two thyme species, *Thymus camphoratus* and *Thymus carnosus*, used in traditional medicine in Portugal, as well as their major compounds was assessed. A special focus was placed on their effect on *Candida albicans* virulence factors. Also, the safety profile of the essential oils was assessed on keratinocytes. The essential oils were analyzed by gas chromatography (GC) and gas chromatography/mass spectroscopy (GC/MS). The minimal inhibitory and minimal fungicidal concentrations of the essential oils and their main compounds were assessed on reference and clinical strains. Also, their effect on *C. albicans* germ tube formation, metabolism, and biofilm disruption were considered. *T. camphoratus* oil was rich in 1,8-cineole and α-pinene whereas *T. carnosus* oil showed high amounts of borneol and camphene. Regarding the antifungal effect, both oils were more active against *Cryptococcus neoformans* and dermatophytes and very effective in inhibiting *C. albicans* germ tube formation, at doses well below their MIC and in a higher extend than the isolated compounds and fluconazole, an antifungal drug widely used in the clinic. The oils also disrupted preformed *C. albicans* biofilms. Furthermore, no toxicity was observed at pharmacological relevant concentrations towards keratinocytes. Our study validates the traditional uses ascribed to these Iberian species. Furthermore, it brings new insights on the antifungal potential and mechanism of action of these thyme species, thus paving the way for the development of novel effective antifungal drugs.

## Introduction

In the last years, the prevalence of fungal infections has increased significantly, especially in immunocompromised individuals, with concomitant life-threatening increments (Richardson and Lass-Flörl, 2008). Additionally, the epidemiology behind systemic fungal infections has changed. Indeed, bloodstream systemic infections (BSI) are mainly caused by *Candida* and *Aspergillus* species being *C. albicans*, *C. glabrata*, *C. parapsilosis*. *C. krusei* and *C. tropicalis* the most common infectiousagents for *Candida*-associated BSI, while *A. fumigatus*, *A. niger*, and *A. flavus* are responsible for the majority of *Aspergillus*-associated BSI (Pfaller et al., 2006). Regarding superficial mycoses, the major causal agents are dermatophytes that remain a health concern worldwide, in both immunocompromised and healthy individuals. This is mainly attributable to the resistance of fungi to conventional antifungal therapies as well as to high relapse rates (Gupta and Cooper, 2008).

For these reasons, fungal infections are still considered a serious problem worldwide that require effective therapeutic strategies. The most common antifungal agents used nowadays include fluconazole, amphotericin B and terbinafine. Nevertheless, these drugs have several reported adverse effects, such as nephrotoxicity and hepatotoxicity. Furthermore, they show a slow response in immunocompromised individuals (Khan and Ahmad, 2011) and have several therapeutic limitations, such as drug interactions, insufficient bioavailability and a fungistatic mechanism of action. In addition, development of resistance as well as innate resistance by emerging species have also been reported (Pfaller, 2012). Therefore, actual antifungal therapies are far from acceptable reinforcing the urgent need for the development of effective antifungal drugs.

Aromatic and medicinal plants, especially those belonging to Lamiaceae and Apiaceae families, have been used for centuries in traditional medicine for the treatment of several pathologies with many therapeutic effects being associated with the presence of volatile compounds, i.e., essential oils. In fact, these compounds have been described as bioactive agents, primarily due to their antifungal, antibacterial and anti-inflammatory properties (Cowan, 1999; Zuzarte et al., 2013; Alves-Silva et al., 2016). In many cases, the use of these plants/compounds is based on empiric knowledge. Thus, the spectrum of action as well as the underlying mechanisms of action are lacking and should be deeply explored.

The genus *Thymus*, one of the most relevant in the Lamiaceae family, includes *ca.* 215 species, many of them endemic to the Mediterranean region (Pirbalouti et al., 2015). Several thyme species are widely used in culinary as flavoring agents and show a pharmacological relevance in traditional medicine being used as spasmolytic, antiseptic, and expectorant agents (Stahl-Biskup, 2003; Pirbalouti et al., 2015). This genus is highly polymorphic regarding the chemical composition of its essential oils, which means that plants morphologically identical can chemically differ, allowing the identification of chemotypes. Indeed, several chemotypes have been identified, for example, in *Thymus vulgaris* (Thompson et al., 2003), *Thymus zygis* subsp. *sylvestris* and subsp. *zygis*, *Thymus mastichina* subsp. *mastichina*, and *Thymus pulegioides* (Figueiredo et al., 2008). The species *T. vulgaris* is one of the most polymorphic species within the genus (Thompson et al., 2003).

Plants from the genus *Thymus* are known for their antifungal properties (Figueiredo et al., 2008). Indeed *T. vulgaris* and *T. zygis* have a European Medicines Agency (EMA) monograph highlighting several effects including their antifungal ones (HMPC/EMA, 2010). Moreover, several thyme species are used in traditional medicine, namely *T. zygis*, *T. mastichina*, *T. camphoratus*, *Thymus carnosus*, and *Thymus caespititius*. These species are often employed for the treatment of infections and inflammation of the mouth, pharynx, skin, and genital tract as well as for the relief of rheumatism (Rivera and Obón, 1995; Carapeto, 2006; Proença da Cunha et al., 2007; Silva et al., 2011). Regarding *T. camphoratus* and *T. carnosus*, both species are traditionally used in Portugal for the treatment of infections of the respiratory tract (dos Santos, 2004; Camejo Rodrigues, 2007). Although some studies on their essential oil composition (Salgueiro et al., 1995, 1997) and biological effects have been reported (Faleiro et al., 2003; Dandlen et al., 2011; Zuzarte et al., 2018), the knowledge regarding the antifungal potential of *T. camphoratus* and *T. carnosus* remains limited. Therefore, the present study was designed to unveil the antifungal activity of these essential oils and their major compounds against yeasts and filamentous fungi and concomitantly to elucidate the mechanisms of action underlying their potential antifungal effect. Importantly, the putative cytotoxicity of the essential oils to human keratinocytes was also assessed since this is a crucial step for further incorporation of these compounds in topical pharmaceutical formulations.

## Materials and Methods

### Plant Material

Flowering aerial parts of *T. camphoratus* Hoffmanns. & Link (Lamiaceae) were collected in Lagos, Algarve, Portugal (N 37° 5′ 53″, W 8° 40′ 4″) whereas those of *T. carnosus* Boiss (Lamiaceae) were collected in Praia da Manta Rota, Algarve, Portugal (N 37° 9′ 49″, W 7° 31′ 7″), during Spring (June). Several plants were obtained to gather a representative sample. Voucher specimens were added to the Herbarium of the University of Coimbra (COI), with accession numbers L. Salgueiro 55 and L. Salgueiro 333 for *T. camphoratus* and *T. carnosus*, respectively. Species authenticity were confirmed by Dr. Jorge Paiva, a taxonomist at the University of Coimbra.

### Essential Oil Isolation and Analysis

The essential oils were obtained by hydrodistillation in a *Clevenger*-type apparatus and analyzed by gas chromatography (GC) and gas chromatography/mass spectrometry (GC/MS) using fused silica capillary columns with two different stationary phases: SPB-1 and SupelcoWax-10, as described by Cavaleiro et al. (2004). Volatile compounds were identified according to their GC retention indices on both SPB-1 and SupelcoWax-10 columns as well as mass spectra. Retention indices, calculated by linear interpolation relative to retention times of C_8_–C_23_ of *n*-alkanes, were compared with those of authentic products included in the Faculty of Pharmacy, University of Coimbra laboratory database (Faculty of Pharmacy, University of Coimbra) and/or literature data (Stein, 2017). Acquired mass spectra were compared with reference spectra from the laboratory database, Wiley/NIST library and literature data (Joulain and König, 1998; Adams, 2007). Relative amounts of individual components were calculated based on GC raw data areas without FID response factor correction.

### Antifungal Activity

#### Fungal Strains

The antifungal activity of the essential oils was evaluated against both reference and clinically isolated strains of several yeasts and filamentous fungi. Yeast strains included *Candida albicans* ATCC 10231, *C. guilliermondii* MAT23, *C. krusei* H9, *C. parapsilosis* ATCC 90018, *C. tropicalis* ATCC 13803, and *Cryptococcus neoformans* CECT 1078; Filamentous fungi namely dermatophyte and *Aspergilllus* strains were selected. Dermatophyte strains included *Epidermophyton floccosum* FF9, *Microsporum canis* FF1, *M. gypseum* CECT 2908, *Trichophyton mentagrophytes* FF7, *T. mentagrophytes* var. *interdigitale* CECT 2958, *T. rubrum* CECT 2794 and *T. verrucosum* CECT 2992 whereas *Aspergillus* strains were *A. flavus* F44, *A. fumigatus* ATCC 46645 and *A. niger* ATCC 16404.

The fungal isolates identified by standard microbiological methods were stored at –80°C on Sabouraud broth with 20% glycerol. Prior to testing, each isolate was inoculated on Sabouraud agar (SDA) to ensure optimal growth and purity.

#### Fungal Growth (MIC and MFC)

Broth macrodilution methods based on the Clinical and Laboratory Standards Institute (CLSI) reference protocols M27-A3 (CLSI, 2008b) and M38-A2 (CLSI, 2008a) for yeasts and filamentous fungi, respectively, were used to determine the minimal inhibitory concentration (MIC) and minimal fungicidal concentration (MFC) of the essential oils and their major compounds (borneol, camphene, 1,8-cineole, α-pinene), as described previously (Zuzarte et al., 2013). MICs were recorded as the lowest concentration of the essential oil/compound that inhibited fungal growth and MFCs were determined as the lowest concentration of the essential oil/compound able to kill the fungal strain. Two reference antifungal compounds, amphotericin B (Fluka, Buchs, Switzerland) and fluconazole (Pfizer, New York, United States), were used to control the sensitivity of the tested microorganisms. The experiments were performed at least in triplicates and a range of values presented whenever the results were not in agreement between replicates.

#### *Candida albicans* Germ Tube Formation

To determine the effect of the essential oils and their major compounds on the yeast-mycelium transition, cell suspensions of *C. albicans* ATCC 10231, from overnight cultures on SDA, were prepared in NYP medium [*N*-acetylglucosamine (Sigma; 10^−3^ mol/L), Yeast Nitrogen Base (Difco; 3.35 g/L), proline (Fluka; 10^−3^ mol/L), NaCl (4.5 g/L), and pH 6.7 ± 0.1 (Marichal et al., 1986)]. The assay was then carried out as previously described by Pinto et al. (2013). Briefly, *C. albicans* suspension was adjusted to a density of 1.0 ± 0.2 × 10^6^ CFU/mL and 990 μL of this suspension was distributed to test tubes. To each tube, 10 μL of sub-inhibitory concentrations of each essential oil and their major compounds were added and the cells were incubated without agitation for 3 h. Then, germ tube formation was registered under a light microscope (40×). Germ tubes were considered when the germinating protuberance was at least as long as the diameter of the blastopore. The conventional antifungal drug, fluconazole, was also tested and DMSO in a maximum concentration of 1% (v/v) was used as a control. The results are presented as the mean ± standard deviation (SD) of three independent experiments.

#### Fungal Viability (MTT Assay)

The effect of the essential oils on *C. albicans* ATCC 10231 viability was determined using the MTT [3-(4,5-dimethylthiazol-2-yl)-2,5-diphenyl-2H-tetrazolium bromide] reduction assay (Pinto et al., 2013) with slight modifications. Briefly, cell suspensions were prepared in RPMI and adjusted to a final density of 0.5–2.5 × 10^3^ CFU/Ml. The suspensions were then distributed into 12-well plates (1 mL/well) and incubated, without agitation, at 37°C. After 18–24 h of incubation, the cells were carefully homogenized, collected and centrifuged at 10,000 rpm for 5 min. Essential oils (0.07–0.57 mg/mL) were added to the pellets in a final volume of 1 mL and then the solutions were transferred to the microwell plate. Following an incubation at 37°C for 1 h, cells were harvested by centrifugation at 10,000 rpm for 5 min and 500 μL of 0.5 mg/mL of MTT (Sigma–Aldrich, St. Louis, MO, United States) in RPMI was added. After an incubation at 37°C for 30 min, the water-insoluble blue formazan crystals (viable cells) were solubilized with 300 μL of DMSO and measured spectrophotometrically at 510 nm using an ELISA microplate reader (SLT, Austria). An additional trypan blue exclusion assay was also performed to confirm the number of living cells.

#### Biofilm Disruption

*Candida albicans* biofilm disruption assays were performed as previously reported (Taweechaisupapong et al., 2012). Briefly, a loop from a *C. albicans* culture in SDA grown for 24 h at 37°C was resuspended in Yeast Peptone Dextrose (YPD) broth (1% yeast extract, 2% peptone, and 2% dextrose) and further incubated for 24 h at 37°C. Then, cells were washed with PBS (pH 7.4) (0.8% NaCl, 0.02% KH_2_PO_4_, 0.31% Na_2_HPO_4_⋅12H_2_O, and 0.02% KCl). Cell density was adjusted to 1 × 10^6^ CFU/mL, and then 100 μL of this suspension was added to 96-well microtiter plates and incubated for 24 h at 37°C to allow biofilm adhesion and formation. Then, the wells were washed 3 times with PBS and the essential oils (0.28–2.2 mg/mL, prepared in RPMI) were added and incubated for 24 h, at 37°C. Essential oil-free wells, with DMSO in a maximum concentration of 1% (v/v), and biofilm-free wells were also included as positive and negative controls, respectively. Biofilm mass was quantified using crystal violet according to Raut et al. (2013). Briefly, after treatments and medium removal, biofilms were fixed with 99% methanol for 15 min. The supernatant was removed, and the wells air-dried. Then, a solution of crystal violet (0.02%) was added to each well and allowed to stain the biofilm for 15 min. Wells were washed 2–3 times with sterile distilled water. Absorbed stain was released by addition of 150 μL of acetic acid (33%) and transferred to fresh wells. The optical density (OD) was read at 620 nm using a microplate reader. The percentage of biofilm formation was calculated by comparing the OD of treated biofilm with that of control biofilm. Biofilm viability was assessed according to the method described by Saharkhiz et al. (2012) with some modifications. Briefly, after the treatments, the medium was removed and 100 μL of a XTT (1 mg/mL in PBS) solution containing 4 μM of menadione (10 mM in acetone) was added to biofilms prewashed with PBS. After 2 h of incubation at 37°C in the dark, the absorbance was read at 490 nm. Biofilm viability was calculated by comparing the absorbance of treated samples with that of positive controls. Results are presented as mean ± SEM of three independent experiments performed in duplicate.

### *In vitro* Cytotoxicity to Keratinocytes (MTT Assay)

The human keratinocytes cell line (HaCaT) was obtained from DKFZ (Heidelberg). Keratinocytes were cultured in Dulbecco’s Modified Eagle Medium (high glucose) supplemented with 100 μg/mL streptomycin and 100 U/mL penicillin, 3.02 g/L sodium bicarbonate and 10% (v/v) inactivated fetal bovine serum, at 37°C in a humidified atmosphere of 95% air and 5% CO_2_. Evaluation of cell viability was performed by the colorimetric MTT assay, as previously reported. Quantification of formazan crystals was performed using an ELISA microplate reader at 570 nm with a reference wavelength of 620 nm. The results were expressed as the percentage of MTT reduction relatively to control cells.

### Statistical Analysis

Data are expressed as mean ± standard error (SEM) of the mean. Statistical significance was determined using one-way analysis of variance (ANOVA), followed by Dunnett’s *post hoc* test. The statistical analysis was performed using Prism 5.0 Software (GraphPad Software). Differences were considered significant for *p* < 0.05.

## Results

### Essential Oil Composition

*Thymus camphoratus* and *T. carnosus* essential oils were obtained with yields of 1.4 and 1.8%, respectively (v/w). The identified volatile compounds accounted for a total amount of 92.5 and 90.1% of the oil for *T. camphoratus* and *T. carnosus*, respectively (Table 1). Both essential oils were characterized by high amounts of oxygen-containing monoterpenes being *T. carnosus* also rich in monoterpene hydrocarbons. The essential oil from *T. camphoratus* was characterized by high amounts of 1,8-cineole (15.5%), α-pinene (12.7%), borneol (8.5%), and camphene (6.6%; Table 1 and Supplementary Figure 1A). *T. carnosus* essential oil was mostly rich in borneol (29.0%), camphene (19.5%), and α-pinene (8.9%; Table 1 and Supplementary Figure 1B).

**Table 1:** Essential oil composition of two Iberian *Thymus* spp.

RI^†^	RI^‡^	Ref RI^x^	Ref RI^#^	Compound	Essential oils	
					*Thymus camphoratus*	*Thymus carnosus*
922	1030	922	1030	α-Thujene	0.5	1.7
928	1025	930	1028	α-Pinene	12.7	8.9
941	1072	943	1073	Camphene	6.6	19.5
959	1447	959	1447	Oct-1-en-3-ol	–	t
962	1253	962	1253	Octan-3-one	–	t
963	1124	964	1126	Sabinene	1.9	1.4
968	1115	969	1116	β-Pinene	0.7	2.1
980	1159	980	1161	Myrcene	0.2	0.1
997	1167	997	1168	α-Phellandrene	0.3	0.1
1008	1187	1010	1187	α-Terpinene	0.6	0.2
1011	1275	1011	1273	*p*-Cymene	0.3	3.5
1019	1204	1020	1205	Limonene	1.5	1.3
1020	1215	1020	1214	β-Phellandrene	–	0.1
1020	1212	1020	1214	1,8-Cineole	15.5	0.4
1025	1235	1025	1235	*Z*-Ocimene	0.1	0.1
1035	1253	1035	1253	*E*-Ocimene	0.9	0.1
1046	1246	1046	1248	γ-Terpinene	0.3	0.9
1050	1459	1051	1459	*E*-Sabinene hydrate	0.2	0.3
1055	1438	1055	1439	*cis-*Linalool oxide	0.2	–
1070	1466	1070	1466	*trans*-Linalool oxide	0.2	–
1077	1284	1077	1288	Terpinolene	0.2	0.1
1082	1542	1080	1544	*Z*-Sabinene hydrate	0.6	2.1
1084	1539	1083	1542	Linalool	5.0	0.2
1106	1488	1104	1487	α-Campholenal	0.4	0.2
1109	n.d.	1106	–	*Z*-p-Menth-2-en-1-ol	t	–
1119	1514	1118	1515	Camphor	4.9	3.5
1122	1645	1119	1649	*E*-Pinocarveol	0.6	0.8
1123	1645	1122	1648	*Z*-Verbenol	0.1	–
1129	1668	1126	1672	*E*-Verbenol	1.1	1.1
1136	1564	1135	1562	Pinocarvone	0.3	–
1145	1691	1146	1695	Borneol	8.5	29.0
1158	1845	1157	1845	*p*-Cymene-8-ol	0.3	0.2
1160	1594	1158	1595	Terpinene-4-ol	4.3	4.9
1164	1622	1165	1621	Myrtenal	0.3	–
1167	1602	1167	1602	Dihydrocarvone	–	0.5
1171	1687	1169	1692	α-Terpineol	0.9	0.5
1177	1698	1177	1698	Verbenone	t	0.1
1178	1780	1177	1786	Myrtenol	0.3	–
1191	1830	1192	1830	*E*-Carveol	0.4	t
1211	1728	1711	1727	Carvone	0.2	–
1214	1679	1214	1679	Neral	–	0.1
1233	1842	1233	1842	Geraniol	0.3	t
1264	1580	1264	1578	Bornyl acetate	3.5	5.2
1268	2183	1267	2183	Thymol	–	0.2
1275	2212	1275	2212	Carvacrol	–	0.1
1324	2159	1325	2163	Eugenol	t	–
1329	1688	1328	1688	α-Terpinyl acetate	0.1	0.2
1342	1455	1342	1455	α-Cubebene	0.2	–
1359	1755	1359	1755	Geranyl acetate	0.8	–
1376	1517	1375	1517	β-Bourbonene	0.1	t
1380	1536	1380	1536	β-Cubebene	0.1	–
1401	1526	1401	1524	α-Gurjunene	–	0.1
1407	1591	1407	1590	*E*-caryophyllene	1.0	0.8
1427	1160	1428	1602	Aromadendrene	0.4	t
1442	1665	1442	1664	α-Humulene	–	t
1447	1636	1447	1637	Alloaromadendrene	–	0.1
1466	1699	1466	1699	Germacrene D	3.6	0.1
1482	1726	1482	1726	Bicyclogermacrene	0.2	–
1495	1723	1495	1723	β-Bisabolene	0.1	–
1502	1752	1498	1752	γ-Cadinene	1.4	0.1
1508	1751	1508	1752	δ-Cadinene	1.1	–
1529	1768	1531	1767	α-Bisabolene	0.5	–
1553	2113	1552	2113	Spathulenol	–	t
1559	1971	1558	1968	Caryophyllene oxide	1.0	0.6
1569	2072	1598	2073	Viridiflorol	–	0.2
1579	2025	1582	2024	Ledol	–	0.1
1594	2089	2596	2091	10-epi-γ-Eudesmol	0.5	t
1607	2158	1606	2158	γ-Eudesmol	–	0.1
1615	2153	1615	2160	τ-Cadinol	2.7	–
1618	2188	1615	2176	α-Muurolol	0.2	–
1618	2043	1620	2052	Cubenol	0.3	–
1622	2215	1622	2215	β-Eudesmol	t	t
1628	2208	1622	2208	α-Eudesmol	–	0.1
1628	2218	1628	2221	α-Cadinol	t	–
1659	2210	1659	2209	α-Bisabolol	t	–
Monoterpene hydrocarbons					26.8	40.1
Oxygen containing monoterpenes					49.1	49.7
Sesquiterpene hydrocarbons					8.7	1.4
Oxygen containing sesquiterpenes					5.4	1.2
Others					0.1	0.1
Total identified					92.5	90.1

*Compounds listed in order to their elution on the SPB-1 column; t = traces ≤0.05%. ^†^Experimental retention indices on the SPB-1 column relative to C_8_–C_24_ n-alkanes. ^‡^Experimental retention indices on the SupelcoWax-10 column relative to C_8_–C_24_ n-alkanes. ^x^Reference retention indices for a polydimethylsiloxane column (Stein, 2017). ^#^Reference retention indices for a polyethylene glycol column (Stein, 2017).*

### Antifungal Activity

#### Fungal Growth (MIC and MFC)

The antifungal activity (MICs and MFCs) of both essential oils and their main isolated compounds is presented in Table 2. Overall, both oils were more effective against *C. neoformans* with MIC values of 0.14 mg/mL and showed poor antifungal activity against the tested *Aspergillus* strains. For *Candida* spp., the essential oils of both species showed a slight fungicidal effect for most of the strains. For dermatophytes, both oils showed a fungicidal effect, but *T. camphoratus* was more effective than *T. carnosus*, presenting lower MIC values for most of the tested strains (Table 2).

**Table 2:** Antifungal activity of *Thymus camphoratus* and *Thymus carnosus* essential oils and their major compounds against pathogenic yeasts, dermatophytes and *Aspergillus* strains.

	*T. camphoratus*		*T. carnosus*		Borneol		Camphene		α-Pinene		1,8-Cineole	
Strains	MIC^†^	MFC^†^	MIC^†^	MFC^†^	MIC^†^	MFC^†^	MIC^†^	MFC^†^	MIC^†^	MFC^†^	MIC^†^	MFC^†^
*Candida albicans* ATCC 10231	1.11–2.23	1.11–2.23	1.11	1.11	2.23	>17.8	>17.8	>17.8	0.57–1.11	0.57–1.11	8.9	8.9
*Candida guilliermondii* MAT23	2.23	2.23	1.11	1.11	2.23	>17.8	>17.8	>17.8	1.11	1.11–2.23	17.8	17.8
*Candida krusei* H9	2.23	2.23	2.23	2.23	4.5	>17.8	>8.9	>17.8	0.14–0.28	0.28	8.9	8.9
*Candida parapsilosis* ATCC 90018	1.11	2.23	2.23	4.5	2.23	2.23	>8.9	>8.9	0.57	0.57	8.9	8.9
*Candida tropicalis* ATCC 13803	4.5	4.5	2.23	2.23	4.5	>17.8	>17.8	>17.8	0.28	0.28	8.9	8.9
*Cryptococcus neoformans* CECT 1078	0.14	0.28	0.14	0.28	1.11	11.1	4.5	4.5	0.07	0.28	4.5–8.9	8.9
*Epidermophyton floccosum* FF9	0.57	0.57	1.11	1.11	2.23	2.23	4.5	4.5	0.14	0.14	4.5	4.5
*Microsporum canis* FF1	0.57	0.57	1.11	1.11	2.23	2.23	4.5	4.5	0.14	0.14–0.28	4.5	4.5
*Microsporum gypseum* CECT 2908	0.57	0.57–1.11	2.23	2.23	2.23	2.23	8.9	8.9	0.14	0.14	4.5–8.9	4.5–8.9
*Trichophyton mentagrophytes* FF7	0.57	0.57	1.11	1.11	2.23	4.5	4.5	4.5–8.9	0.28	0.28–0.57	4.5	4.5
*Trichophyton mentagrophytes* var. *interdigitale* CECT 2958	1.11	1.11	1.11	2.23	2.23	4.5	8.9–17.8	8.9–17.8	0.28	0.28	8.9	8.9
*Trichophyton verrucosum* CECT 2992	1.11	1.11	2.23	2.23	2.23	2.23	17.8	17.8	1.11	1.11	8.9	8.9–17.8
*Trichophyton rubrum* CECT 2794	0.57	0.57	0.57	1.11	2.23	2.23	2.23–4.5	4.5	0.07	0.07	2.23–4.5	4.5
*Aspergillus flavus* F44	4.5	>8.9	4.5	>8.9	4.5	>17.8	>17.8	>17.8	1.11	1.11	17.8	17.8
*Aspergillus fumigatus* ATCC 46645	2.23	>8.9	2.23	8.9	2.23	>17.8	>17.8	>17.8	1.1	1.11–223	8.9	8.9–17.8
*Aspergillus niger* ATCC 16404	2.23	>8.9	2.23	>17.8	4.5	>17.8	>17.8	>17.8	2.23	4.5	8.9	>17.8

*^†^MIC and MFC were determined by a macrodilution method and expressed in mg/mL. Results were obtained from 3 independent experiments performed in duplicate.*

Regarding the antifungal activity of the isolated compounds, α-pinene was, by far, the most effective compound (Table 2). Indeed, α-pinene showed a fungicidal effect for most of the tested strains, being very effective against *C. neoformans* and *T. rubrum* (MIC = 0.07 mg/mL). Contrarily, 1,8-cineole and camphene were ineffective against many of the tested strains, while borneol showed a slight antifungal effect (MIC ranging from 1.11 to 4.5 mg/mL; Table 2).

#### Germ Tube Inhibition in *Candida albicans*

Since the yeast-to-hypha transition is an important virulence factor associated with the pathogenesis of *C. albicans* infections, the effect of the oils on the germ tube formation in *C. albicans* ATCC 10231 was assessed. As shown in Table 3, both essential oils inhibited the germ tube formation at concentrations well below their respective MIC, with *T. camphoratus* being more effective and attaining ca. 40% inhibition at 0.07 mg/mL (MIC/16). Fluconazole (MIC = 0.001 mg/mL), the antifungal drug of choice in the clinic for the management of candidiasis, failed to inhibit this feature even at concentrations much higher than its MIC. Indeed, even at 0.2 mg/mL, that corresponds to fluconazole’s MIC×200 (Table 4), the antifungal drug was completely ineffective.

**Table 3:** Effect of sub-inhibitory concentrations of the essential oil of *Thymus camphoratus*, *Thymus carnosus* and their major compounds on the germ tube formation of *Candida albicans* ATCC 10231.

	*T. camphoratus*	*T. carnosus*	α-Pinene	Borneol	1,8-Cineole	Camphene

Control^†^	100	100	100	100	100	100
0.02^‡^	91.7 ± 3.0^∗∗^	98.1 ± 1.7	–	–	–	–
0.04	81.9 ± 1.2^∗∗∗∗^	90.3 ± 2.2	93.2 ± 0.8	93.1 ± 2.3	–	–
0.07	62.0 ± 2.1^∗∗∗∗^	79.7 ± 2.8^∗∗∗^	86.3 ± 0.7^∗^	89.2 ± 2.5	–	–
0.14	43.1 ± 5.3^∗∗∗∗^	47.1 ± 4.9^∗∗∗∗^	55.7 ± 7.0^∗∗∗∗^	76.6 ± 4.3^∗∗∗∗^	–	–
0.28	11.3 ± 2.2^∗∗∗∗^	7.3 ± 2.6^∗∗∗∗^	4.9 ± 1.8^∗∗∗∗^	58.7 ± 4.0^∗∗∗∗^	85.7 ± 7.1	91.8 ± 0.5
0.57	0.0 ± 0.0^∗∗∗∗^	0.0 ± 0.0^∗∗∗∗^	0.0 ± 0.0^∗∗∗∗^	29.6 ± 2.1^∗∗∗∗^	66.7 ± 1.2^∗∗^	74.5 ± 7.3
1.11	–	–	–	4.5 ± 0.9^∗∗∗∗^	29.4 ± 3.2^∗∗∗^	48.4 ± 12.9^∗^
2.23	–	–	–	–	1.2 ± 0.1^∗∗∗∗^	10.0 ± 6.6^∗∗∗∗^
4.5	–	–	–	–	–	1.9 ± 1.9^∗∗∗∗^
8.9	–	–	–	–	–	0.0 ± 0.0^∗∗∗∗^

*^†^Samples without essential oil, with 1% (v/v) DMSO. ^‡^Absolute concentration of essential oil in mg/mL. Results are means ± SEM of a minimum of three independent experiments; ^∗^p < 0.05, ^∗∗^p < 0.01, ^∗∗∗^p < 0.001, ^∗∗∗∗^p < 0.0001, compared to control (100%).*

**Table 4:** Effect of sub-inhibitory concentrations of fluconazole on the germ tube formation of *Candida albicans* ATCC 10231.

	*Fluconazole*

Control^†^	100
0.200^‡^	89.10 ± 5.09
0.128	90.04 ± 9.94
0.004	98.86 ± 6.07
0.002	100.53 ± 7.81
0.001	101.00 ± 5.27

*^†^Sample without fluconazole, with 1% (v/v) DMSO. ^‡^Absolute concentration of fluconazole in mg/mL. Results are means ± SEM of a minimum of three independent experiments.*

The effect of the isolated major compounds, namely borneol, camphene, 1,8-cineole and α-pinene was also considered to assess which of these compounds could contribute to the activity of the oils. Our results show that both essential oils have a stronger effect on the inhibition of the germ tube formation in comparison to the isolated major compounds. Remarkably, although α-pinene showed lower MIC and MFC values than the essential oil (Table 2), our results demonstrate that both essential oils affect *C. albicans* germ tube formation at lower concentrations than α-pinene (Table 3).

#### *Candida albicans* Viability

**FIGURE 1:**
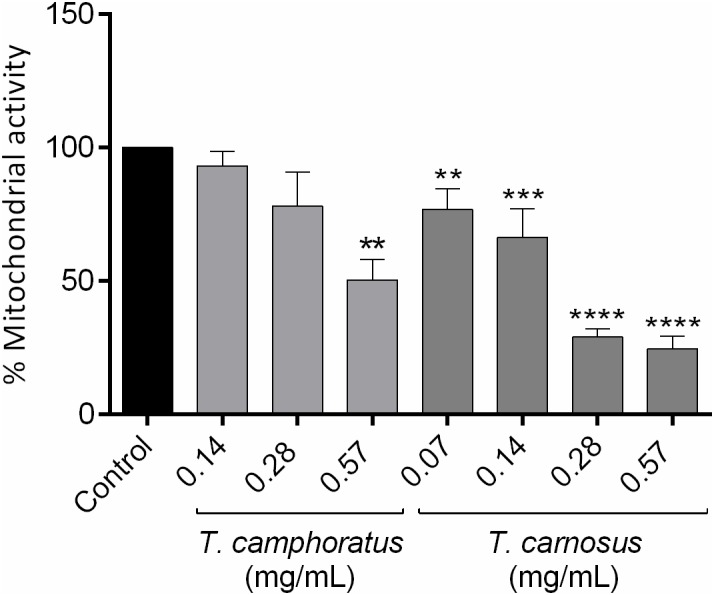
Viability of *C. albicans* ATCC 10231 cells treated with different concentrations of *Thymus camphoratus* and *Thymus carnosus* essential oils. One-way ANOVA was performed separately for each species and values are expressed as % of mitochondrial activity relative to control (mean ± SEM of three independent assays). ^∗∗^*p* < 0.01, ^∗∗∗^*p* < 0.001, ^∗∗∗∗^*p* < 0.0001, compared to control.

The effect of the essential oils on *C. albicans* viability was also assessed (Figure 1). Once again, our results show that both oils inhibited *C. albicans* mitochondrial activity at concentrations lower than their respective MIC. Nevertheless, *T. carnosus* essential oil showed better results since it significantly reduced *C. albicans* mitochondrial activity at concentrations ranging from 0.07 to 0.57 mg/mL. Indeed, a viability decrease of more than 70% was observed at 0.28 mg/mL for *T. carnosus* essential oil whereas *T. camphoratus* oil decreased cell viability of the yeast only *ca*. 44% at 0.57 mg/mL and at lower concentrations (≤0.28 mg/mL) was ineffective (Figure 1). A trypan blue exclusion assay, that assesses the integrity of the cell wall, showed 100% of living fungi cells in all the tested concentrations (data not shown), thus confirming the adverse effect on the essential oils directly on mitochondrial activity.

#### Disruption of Preformed Biofilms in *Candida albicans*

**FIGURE 2:**
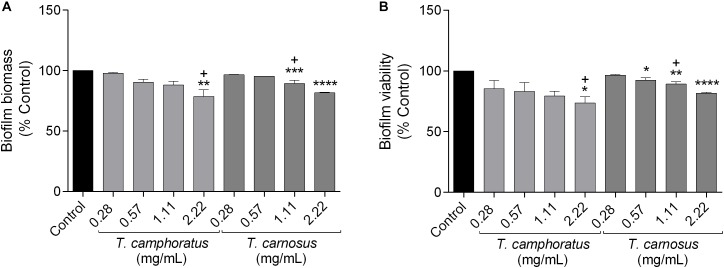
*Candida albicans* ATCC 10231 biofilm biomass **(A)** and biofilm viability **(B)**. Cells were treated with different concentrations of *Thymus camphoratus* and *Thymus carnosus* essential oil. Biofilm mass was quantified by the crystal violet assay and biofilm viability assessed using the XTT assay. One-way ANOVA was performed separately for each species and values are expressed as a percentage of biofilm biomass or viability relative to control (mean ± SEM of three independent assays). ^∗^*p* < 0.05, ^∗∗^*p* < 0.01, ^∗∗∗^*p* < 0.001, ^∗∗∗∗^*p* < 0.0001, compared to control. “+” indicates MIC values of the essential oils.

Bearing in mind that *C. albicans* biofilms are also important virulence factors, the capacity of the essential oils to decrease both biofilm mass (Figure 2A) and biofilm viability (Figure 2B) were also assessed. Overall, both oils were effective at concentrations close to their respective MIC (Figure 2), with *T. carnosus* essential oil being slightly more active in reducing biofilm viability since it decreased this feature at MIC/2 (0.57 mg/mL; Figure 2B). Interestingly, fluconazole was ineffective in decreasing biofilm biomass showing a slight effect in biofilm viability reduction at concentrations much higher than its MIC (MIC×128; data not shown).

### Cytotoxicity of the Essential Oils

*Thymus carnosu*s essential oil was slightly more toxic (IC_50_ = 0.705 mg/mL) than *T. camphoratus* oil (IC_50_ = 0.848 mg/mL). Nevertheless, at bioactive concentrations, against the majority of the dermatophyte strains, as well as at concentrations that inhibited *C. albicans* germ tube formation, no toxicity was observed, thus pointing out a safety profile for a further development of topical formulations (Table 5). Additionally, at lower concentrations (below 0.28 mg/mL) both essential oils increased cell viability (Table 5). This could be due to an increase in keratinocytes proliferation or due to the stimulation of their mitochondrial activity and therefore, additional studies should be conducted to further understand this event.

**Table 5:** Effect *of Thymus camphoratus* and *Thymus carnosus* essential oils on keratinocytes viability.

Essential oil	Concentration (mg/mL)	Keratinocytes HaCaT (% of control)
*Thymus camphoratus* (IC_50_ = 0.953 mg/mL)	1.11	54.03 ± 8.42
	0.57	96.81 ± 4.91
	0.28	127.2 ± 25.62
	0.14	146.8 ± 29.70
	0.07	134.6 ± 20.33
*Thymus carnosus* (IC_50_ = 0.793 mg/mL)	1.11	44.05 ± 4.48^∗^
	0.57	82.56 ± 1.31
	0.28	103.2 ± 18.94
	0.14	107.4 ± 13.71
	0.07	122.6 ± 21.88

*Results are expressed as percentage of MTT reduction relatively to control cells maintained in culture medium. Each value represents mean ± SEM of at least three independent experiments performed in duplicate. Statistical differences compared to control cells (100%). ^∗^p < 0.05, using a one-way ANOVA followed by a Dunnett’s multiple comparison test.*

## Discussion

Previous studies on the chemical composition of *T. camphoratus* and *T. carnosus* essential oils reported some chemical variability,as frequently shown for other thyme species. For example, Salgueiro et al. (1997) studied different populations of *T. camphoratus* collected in Algarve and Baixo Alentejo (south of Portugal) and showed a high chemical variability, particularly among individual samples or small populations, being α-pinene (7.0–11.0%), borneol (1.0–24.0%), and 1,8-cineole (3.9–20.0%) the major compounds identified. In another study, the essential oils from both flowers and leaves of *T. camphoratus* collected in Algarve, Portugal, showed very high amounts of 1,8-cineole, 56.7 and 62.7% for flowers and leaves, respectively (Faleiro et al., 2003). Similarly, for *T. carnosus* of different regions of Algarve and Baixo Alentejo, Portugal, a chemical variability was observed between the samples. Nevertheless, most of the essential oils were characterized by high amounts of borneol (23.0–32.0%), camphene (6.0–13.0%), terpinen-4-ol (4.0–12.2%), and *cis*-sabinene hydrate (2.5–10.7%) (Salgueiro et al., 1995). In our study, plants growing in large populations of the Algarve region (Portugal) were collected for essential oil extraction, therefore, the chemical profile described is the most prevalent in *T. carnosus* and *T. camphoratus* growing in the south of Portugal. Furthermore, Stahl-Biskup (Stahl-Biskup, 2003) described that thymol and carvacrol, both terpene phenols, as well as their biogenetic derivatives are important compounds in the genus *Thymus*, followed by linalool, borneol, terpinen-4-ol, and 1,8-cineole. Indeed, thymes rich in 1,8-cineole, such as *T. mastichina* have a very high industrial interest, having an ISO monograph (ISO 4728:2003, 2015), thus highlighting the potential industrial interest for the species herein described.

Studies on the antifungal activity of *T. camphoratus* and *T. carnosus* essential oils are scarce and based upon different methodologies. Indeed, previous studies using the disk diffusion method, showed a weak or even no inhibitory effect of both oils on *C. albicans* (Faleiro et al., 2003; Dandlen et al., 2011). Regarding the antifungal potential of other thyme species with high amounts of 1,8-cineole, some studies have been reported. For example, the essential oils of *T. mastichina* and *T. capitellatus* (Pina-Vaz et al., 2004; Salgueiro et al., 2006) showed a weaker antifungal effect against several yeasts, dermatophytes and *Aspergillus* strains compared with the oils tested in the present study. In opposition, the essential oil from *T. herba-barona* (Zuzarte et al., 2013)*, T. zygis* subsp. *sylvestris* (Pina-Vaz et al., 2004), *T. zygis* subsp. *zygis* (Gonçalves et al., 2010), and *T. vulgaris* (Pina-Vaz et al., 2004) showed a more preeminent activity due to their high content in phenolic terpenoids, namely thymol and/or carvacrol. In the present study, the antifungal effect of the essential oils could be mainly attributed to α-pinene since it was found in considerable amounts in both oils. Nevertheless, active minor compounds may also contribute to the activity of the essential oil. For example, linalool, cadinol, and terpinen-4-ol, all minor compounds of both *T. carnosus* and *T. camphoratus* essential oils, have also been widely described as antifungal agents (Pinto et al., 2013; Su and Ho, 2016) and an anti-yeast addictive effect has been reported for α-pinene and limonene (Tserennadmid et al., 2011).

To the best of our knowledge, the present study is the first report on the effect of *T. camphoratus* and *T. carnosus* essential oils on *C. albicans* germ tube formation. Assessing this feature is of great relevance since the yeast-to-hypha transition represents the main virulence factor associated with candidosis. Also, filamentation is essential for the development of robust biofilms, another major virulence factor associated with the pathogenesis of *C. albicans* (Romo et al., 2017). Indeed, fluconazole, the main antifungal used in the clinic, is ineffective on germ tube inhibition. Our findings show that very low doses of the essential oils are able to inhibit filamentation in *C. albicans*, being more effective than the isolated compounds tested. The stronger effect of the essential oils may be explained by the presence of active minor compounds. Indeed, linalool, a minor compound in both oils has been described as an effective natural compound for inhibiting germ tube formation in *C. albicans* (Hsu et al., 2013). Regarding other thyme species, few studies have demonstrated inhibitory effects, but with variable results between species. Interestingly, although phenolic thyme essential oils, such as those from *T. vulgaris* and *T. zygis*, show potent antifungal activities they are much less effective in inhibiting *C. albicans* germ tube formation than *T. camphoratus* and *T. carnosus* oils. Indeed *T. vulgaris* and *T. zygis* essential oils were only able to achieve significant inhibitions of filamentation (over 50%) at MIC values (0.16–0.32 μL/mL) (Pina-Vaz et al., 2004), whereas *T. camphoratus* essential oil attained about 40% of inhibition at much lower concentrations (0.07 mg/mL) and at MIC/4, ca. 90% of inhibition was observed (Table 3).

Regarding *C. albicans* viability, once again, as far as it is known, the present study is the first to report the effect of *T. camphoratus* and *T. carnosus* oils on *C. albicans* mitochondrial activity. Previously, other thyme essential oils were tested, namely *T. villosus* subsp. *lusitanicus*. Nevertheless, contrarily to our results, only detrimental effects were observed at concentrations close to the respective MIC (Pinto et al., 2013). Moreover, both *T. camphoratus* and *T. carnosus* essential oils were effective in disrupting *C. albicans* biofilms, being *T. carnosus* oil slightly more active in reducing yeast viability. It is well known that, similarly to bacteria, fungi in biofilms show a higher resistance toward antifungals than planktonic fungi, due to the expression of several resistance genes and phenotypic modifications. Indeed, *C. albicans* biofilms have been reported as highly resistant to fluconazole (Uppuluri et al., 2011). Therefore, the disruption of this highly organized system is a very attractive target for the development of effective antifungals (Alves-Silva et al., 2016). It is important to emphasize that studies evaluating the effect of thyme essential oils on preformed biofilms are lacking. Nevertheless, regarding isolated compounds, several terpenes found in *T. carnosus* and *T. camphoratus* essential oil have been previously tested. Some compounds such as 1,8-cineole showed an inhibitory effect at high concentrations, 8 mg/mL (Hendry et al., 2009). Moreover, several other compounds showed an inhibitory effect on the biofilm formation, namely camphor, camphene, and borneol (Manoharan et al., 2017) as well as α-pinene (Silva et al., 2012). Furthermore, linalool, a minor compound found in both oils, was able to disrupt preformed *C. albicans* biofilms (Hsu et al., 2013; Raut et al., 2013) and terpinen-4-ol was able to disrupt preformed single and dual-species *Candida* biofilms in a dose-dependent manner (Francisconi et al., 2015).

Taken together these results show that *T. camphoratus* and *T. carnosus* essential oils have anti-virulent potential, thus preventing disseminative candidosis.

Bearing in mind a future topical application that is more amenable and shows fewer side effects than oral or intranasal applications, the effect of the essential oil on keratinocytes was assessed, with no toxicity observed at pharmacological relevant concentrations.

## Conclusion

This study highlights the antifungal potential of two Iberian endemic thyme species: *T. camphoratus* and *T. carnosus*. Of relevance, the results demonstrated that both oils were able to inhibit the growth of *Cryptococcus neoformans* and several dermatophyte strains. Moreover, the oils were very effective in inhibiting *C. albicans* germ tube formation at concentrations well below the MIC and in a much higher extend than fluconazole, an antifungal drug widely used in the clinic. Finally, *T. carnosus* oil was more effective in decreasing yeast mitochondrial activity and disrupting preformed biofilms in *C. albicans*. Overall, our results point out promising anti-virulent effects for these oils, foreseeing a potential application in the management of disseminative candidiasis.

Our findings add relevant information to the pharmacological activity of these species concomitantly providing new insights to their mechanism of action and reinforcing the use of thyme plants as topical antiseptics and disinfectants, since toxicity towards keratinocytes was absent at most of the bioactive concentrations. In addition to the relevant antifungal activity, both thymes have a very good essential oil yield, which increases their commercial interest and industrial potential. Overall, our results raise awareness on species poorly recognized and valued, thus paving the way for a better industrial exploitation of these plants namely in the pharmaceutical field.

## Author Contributions

MA and MG performed the experiments that originated this work and analyzed the data. MZ and JA-S made the literature review and wrote the manuscript. CC carried out the chemical characterization of the essential oils. CC and MC reviewed the manuscript. LS supervised the work and reviewed the manuscript.

## Conflict of Interest Statement

The authors declare that the research was conducted in the absence of any commercial or financial relationships that could be construed as a potential conflict of interest.
