# Supplementary material for: Unveiling the Antifungal Potential of Two Iberian Thyme Essential Oils: Effect on C. albicans Germ Tube and Preformed Biofilms
Source: Front Pharmacol. 2019 May 2;10:446. doi: 10.3389/fphar.2019.00446 (PMC6509473; doi:10.3389/fphar.2019.00446)
Supplement: Supplementary file 1 [file Data_Sheet_1.PDF]

Supplementary Material

Unveiling the antifungal potential of two Iberian thyme essential oils: effect on *C. albicans* germ tube and preformed biofilms

Melissa Alves[1,*], Maria José Gonçalves[1,*], Jorge M. Alves-Silva[1,2], Mónica Zuzarte[2], Carlos Cavaleiro[1], Maria Teresa Cruz[3], Lígia Salgueiro[1,#]

* These authors contributed equally to the work.

[#] **Correspondence:** Lígia Salgueiro: ligia@ff.uc.pt

1 Supplementary Figures and Tables

1.1 Supplementary Figures

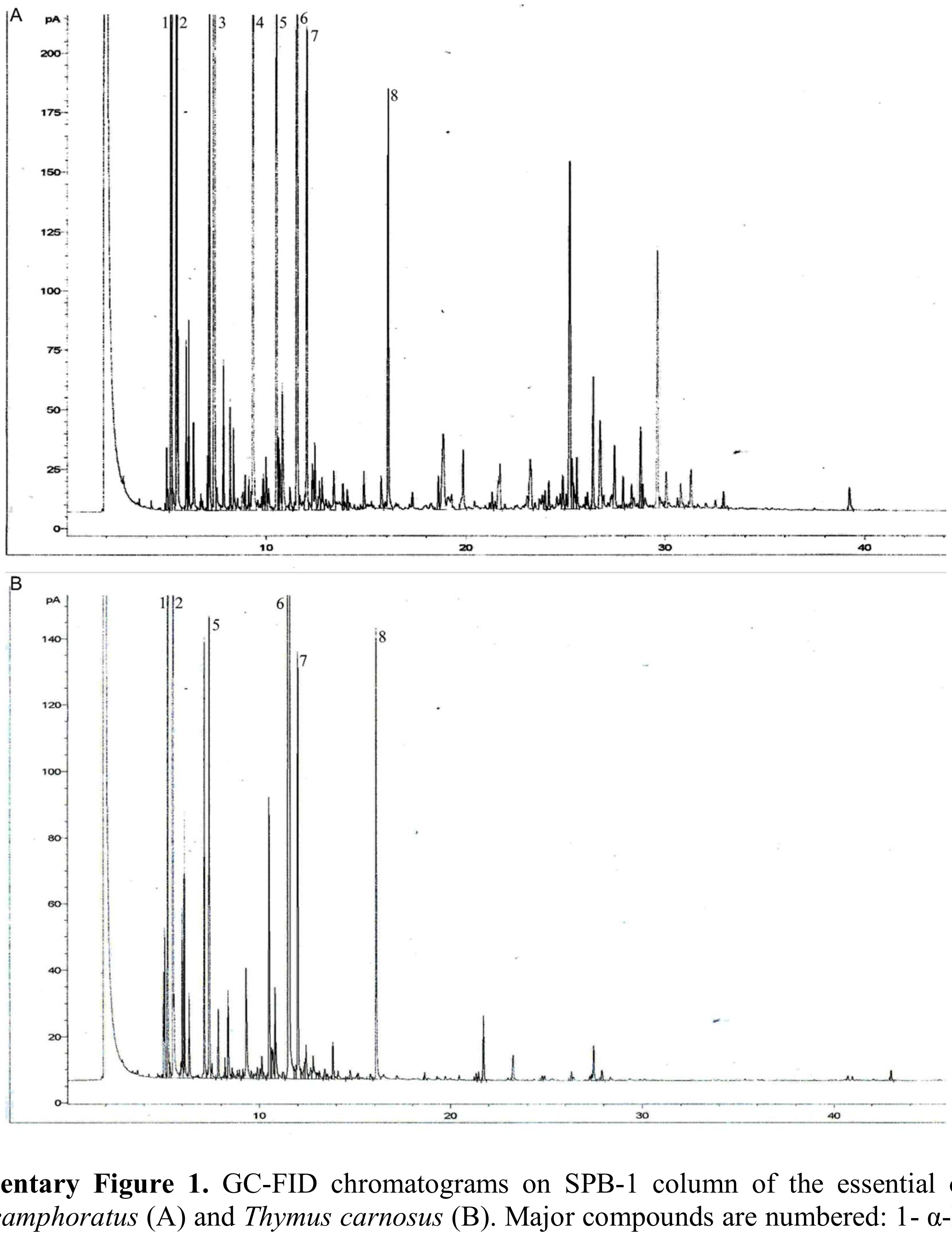

Supplementary Figure 1. GC-FID chromatograms on SPB-1 column of the essential oils from *Thymus camphoratus* (A) and *Thymus carnosus* (B). Major compounds are numbered: 1- α-Pinene; 2 – Camphene, 3 – 1,8-Cineole; 4 – Linalool; 5 – Camphor; 6 – Borneol; 7 – Terpinene-4-ol; 8 – Bornyl acetate
